# Relationship of Catheter Contact Angle and Contact Force with Contact Area on the Surface of Heart Muscle Tissue in Cardiac Catheter Ablation

**DOI:** 10.1007/s13239-021-00529-8

**Published:** 2021-03-15

**Authors:** Kriengsak Masnok, Nobuo Watanabe

**Affiliations:** 1 Functional Control Systems, Graduate School of Engineering and Science, Shibaura Institute of Technology, Saitama, Japan; 2 Department of Bio-Science and Engineering, College of Systems Engineering and Science, Shibaura Institute of Technology, Saitama, Japan

**Keywords:** Catheter ablation, Contact force, Contact angle, Contact area, Tachyarrhythmia

## Abstract

**Purpose:**

The aims of this study were to develop an experimental procedure for setting the catheter angle with respect to the surface of the heart muscle and the catheter contact force and to investigate the catheter contact area on the heart muscle as a function of catheter contact angle and force.

**Methods:**

Visualization tests were performed for 5 contact angles (0°, 30°, 45°, 60°, and 90°) and 8 contact forces (2, 4, 6, 10, 15, 20, 30, and 40 gf). Each experiment was repeated 6 times with 2 different commercially available catheter tips.

**Results:**

The morphology of the contact area was classified into rectangular, circular, ellipsoidal, and semi-ellipsoidal. The correlation between contact force and contact area was a logarithmic function; increasing contact force was associated with increased contact area. At the same contact force, the correlation between contact angle and contact area was inverse; decreasing contact angle was associated with a corresponding increase in contact area.

**Conclusion:**

Both the catheter contact angle and contact force substantially impact the contact area and morphology in catheter ablation procedures.

## Introduction

Over the past three decades, cardiac catheter ablation therapy has become a widely used and effective treatment for tachyarrhythmia.^7^,^9^,^10^,^14^,^24^,^31^ In this treatment, radiofrequency current is applied to the heart, heating the target area to a temperature exceeding 50 °C through resistive heating,^19^ thereby creating a lesion that isolates the abnormal electric pathway.^8^,^17^ Earlier studies have revealed several factors that correlate with lesion size, evaluated in terms of ablated area, volume, and depth.^2^,^18^,^28^,^30^ These factors include ablation circuit impedance,^8^,^30^ electrical power,^16^,^21^,^25^ energy delivery,^11^,^13^ catheter diameter,^15^,^26^ exposure time,^8^,^16^ contact force,^1^,^5^,^16^,^21^,^22^,^30^ ablation electrode temperature,^12^,^29^ irrigation saline flow amount,^5^,^25^,^28^ and blood flow near the myocardial surface.^6^,^11^,^20^,^23^ Among these factors, catheter contact force is reported to show a strongly positive correlation with lesion size.^1^,^3^–^5^,^22^,^27^

In addition to these factors, we hypothesized that the catheter contact angle with respect to the surface of the heart muscle would also have a substantial effect. During radiofrequency current catheter ablation, the catheter tip should contact the heart tissue surface at a variety of angles. However, no studies to date have investigated the relationship between the catheter tip and contact area with the heart muscle.

Against this backdrop, the purpose of this study was to develop an experimental procedure for setting the catheter angle with respect to the surface of the heart muscle and the catheter contact force, as well as to investigate the catheter contact area on the heart muscle as a function of catheter contact angle and contact force.

## Methods

### Heart Muscle Surface Flattener and Different Open-Loop Irrigated Catheter Tips

Most of the surface of the heart is round, and the state of catheter contact would vary according to clinical conditions. Therefore, to provide better reproducibility of our *in vitro* experiments, we developed a special instrument that precisely adjusts the catheter angle between the catheter tip and the heart muscle.

The instrument consists of a heart muscle surface flattener and catheter tip angle setter. As part of the heart muscle surface flattener, a circular crystalline acrylic plate with a thickness of 12 mm and a diameter of 130 mm was used to flatten the surface of porcine heart tissue and fix its position at a specific location and orientation, ensuring that all experiments using this plate will maintain uniformity.

**Figure 1:**
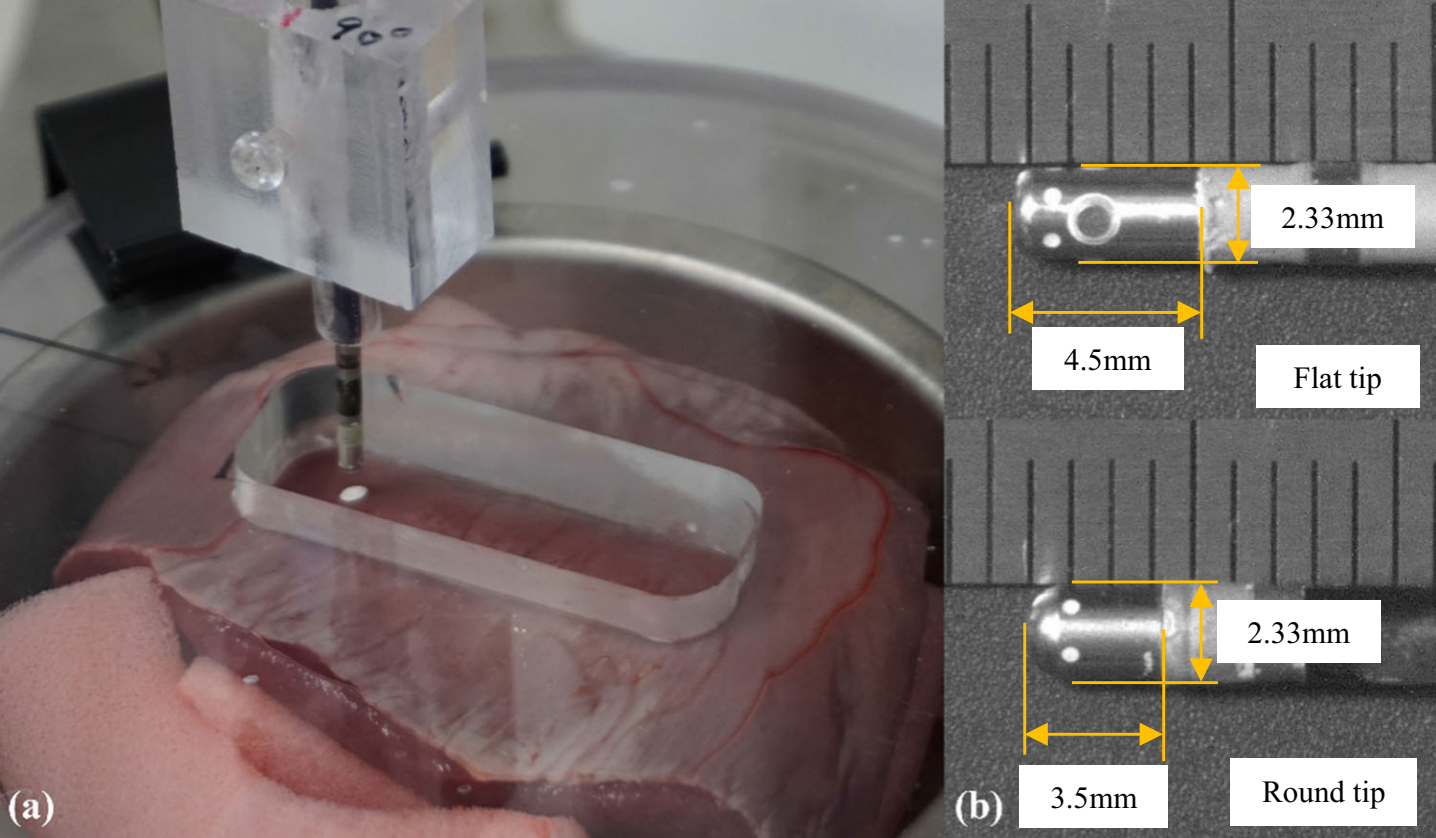
Heart muscle surface flattener and shape of the catheter tip; (**a**) photograph of the heart muscle surface flattener. The heart muscle tissue is sandwiched between a flat acrylic plate and a soft sponge, which are placed in a stainless bowl, and the surface of the heart muscle was flattened by adjusting the amount of the sponge in the bottom of the bowl. (**b**) Ablation catheter tips with two shapes were used: flat (top) and round (bottom).

The porcine heart was obtained from a slaughterhouse, cut into pieces, and stored in a refrigerator. Before the experiment, the pieces were removed from the refrigerator and kept at room temperature under moist conditions to prevent drying. The porcine heart tissue was sandwiched between the acrylic plate and a soft sponge placed in a stainless bowl. The surface of the heart muscle surface was flattened by adjusting the amount of the sponge. The catheter contact experiments were performed through a hole (20 mm × 50 mm) in the acrylic plate, as shown in Fig. 1a. All experiments were performed within 3 days after the pig was sacrificed at the slaughterhouse.

Two different open-loop irrigated catheter tips were used in this study (Fig. 1b). One was the IntellaNav Mifi™ OI (7 Fr/4.5 mm 7.5 Fr; PMR9620, Boston Scientific, Inc.; top of Fig. 1b), which is representative of flat-tip catheters. It was 110 cm long, with a tip length of 2.33 mm, and had a standard curve style. The other catheter was an Abbott TactiCath™ (7 Fr/3.5 mm 7.5 Fr; Quadripolar, PN-004075, St. Jude Medical, Inc.; bottom of Fig. 1b), which is representative of round-tip catheters. It was 115 cm long, with a tip length of 2.33 mm, and had a steerable curve style. Both catheters were open-loop irrigated catheters, with six small irrigation holes circumferentially located on the lateral surface of the tip. Irrigation of the catheter tip was designed to reduce excessive heating of the tissue and blood at the catheter tip. The main difference between the two catheters is the shape of the end tip.

### Experimental Procedure and Evaluation

**Figure 2:**
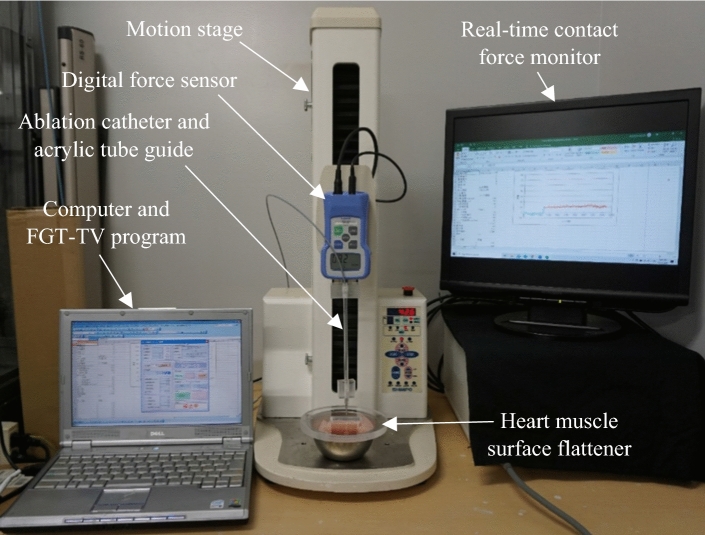
Experimental setup. A compact desktop test stand equipped with a digital force gauge was controlled using an FGT-TV software link from a computer. In this picture, the acrylic tube guide for 90° was used.

To elucidate the effects of the catheter contact angle and contact force on the contact area of the heart tissue surface, we developed a special experimental procedure that enables the setting of various catheter contact angles (0°, 30°, 45°, 60°, and 90°) using a special acrylic tube guide, as well as the measurement of the contact force. In the experimental setup, a digital force sensor (FGP-0.5, Nidec-Shimpo Corporation) was mounted on a motion stage (FGS-5000TV, Nidec-Shimpo Corporation), the position of which can be controlled vertically. Using this setup, the catheter contact force and contact angle could be precisely controlled. The system was operated using commercial software (FGT-TV) running on a computer, as shown in Fig. 2.

White soluble ink (Pen Cure, Japan Pen Company) was overlaid on the metal electrode of the catheter tip to visualize the contacted area on the heart tissue surface. Then, the 8 levels of contact force within the clinically used range (2, 4, 6, 10, 15, 20, 30, and 40 gf) were applied to the cardiac muscle in line with the typical clinical contact force ranges.^3^,^4^,^27^ Using this process, the catheter contact area-visualization test was repeated 6 times each for the 5 contact angles and 8 contact forces to ensure equal distribution of contact force. In the final step, all of the catheter contact areas for each condition were photographed for the evaluation of contact area through image analysis. In total, 480 experiments were performed.

### Morphological Evaluation of Catheter Contact Area

Image analysis of photographs of the catheter contact area was performed to evaluate the morphology of the contact area. The image analysis program MATLAB (Version 2019a) was used to perform the following actions. First, the raw color image of the contact area was manually segmented into individual lesion images and converted into a grayscale image, and finally the grayscale image was binarized. Next, the catheter contact area on the heart tissue surface was calculated. To understand the morphology of the contact area, the centroid of each contact area image was aligned to create a reference point for comparison. Then, the image was rotated about the centroid to align each area's longest axis parallel to the vertical direction. The average morphology of the contact area was derived from six experimentally acquired images. The morphological characteristics corresponding to physical parameters were also evaluated. All statistical analyses were performed using GraphPad Prism software (Version 8.4.3).

## Results

### Contact Area Morphology

**Figure 3:**
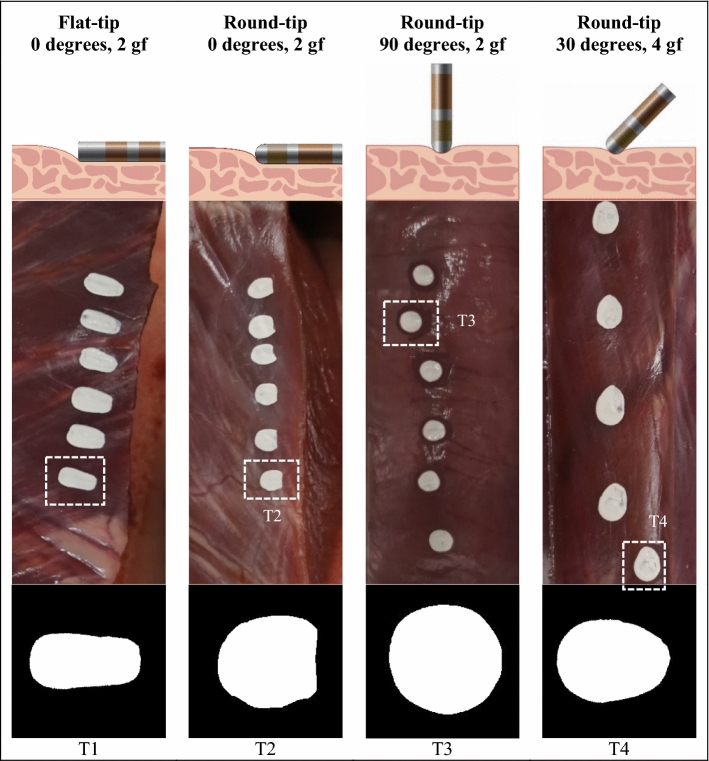
Morphology of the contact area on a porcine heart under various contact conditions. T1–T4 represent the morphology of the four types of contact area.

Figure 3 shows the four distinct morphologies of the contact area with a contact angle of 0° using the flat-tip catheter and contact angles of 0°, 30°, and 90° using the round-tip catheter. These images show that the contact angle and shape of the catheter tip can affect the contact area morphology. For example, the morphology differs according to the shape of the catheter tip, even when both are applied at a contact force of 2 gf and a contact angle at 0°. In contrast, the morphology was similar when both shapes were applied at a contact force of 2 gf and a contact angle at 90. Further details about the differences in contact area morphology will be discussed later in the “Discussion” section.

### Average Contact Area

**Figure 4:**
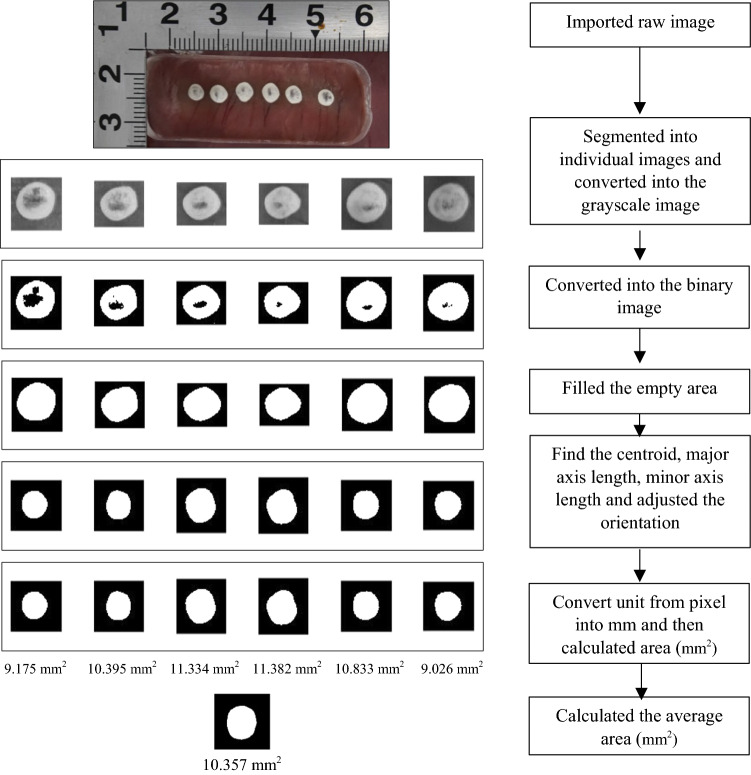
Chart of the flow process for evaluating the morphology of the contact area and the average contact area at a contact angle of 90° and a contact force of 30 gf using a round-tip catheter.

Figure 4 shows the image analysis flow process for evaluating the morphology of the contact area and the average contact area on a porcine heart with a contact angle of 90° and a contact force of 30 gf using the round-tip catheter.

Data of average contact areas, standard deviations, and percentage of contact area for flat-tip and round-tip catheters are shown in Tables 1 and 2, respectively. The ratio of the area of the catheter in contact with heart muscle to the catheter tip surface area was calculated by the following equation:

**Table 1:** Average contact area and percentage contact area when using the flat-tip catheter (mm^2^).

Contact angle	Contact force (gf)											
	2			4			6			10		
	AVG CA	SD	PCA (%)	AVG CA	SD	PCA (%)	AVG CA	SD	PCA (%)	AVG CA	SD	PCA (%)
0°	11.424	1.123	31	10.861	1.188	29	10.547	1.079	28	14.041	1.245	38
30°	6.002	0.908	16	7.406	0.594	20	7.941	1.625	21	9.607	1.284	26
45°	7.058	0.928	19	7.027	0.608	19	10.199	0.873	27	10.463	0.883	28
60°	4.641	0.605	12	7.247	1.228	19	8.163	1.791	22	10.094	1.996	27
90°	3.445	0.831	9	4.392	0.514	12	5.627	0.555	15	6.879	0.874	18

Contact angle	Contact force (gf)											
	15			20			30			40		
	AVG CA	SD	PCA (%)	AVG CA	SD	PCA (%)	AVG CA	SD	PCA (%)	AVG CA	SD	PCA (%)
0°	15.458	1.392	41	15.358	1.624	41	16.405	1.050	44	19.097	1.294	51
30°	13.178	2.103	35	14.644	2.609	39	11.769	1.914	32	14.759	1.196	40
45°	11.820	0.680	32	12.842	1.438	34	14.860	1.672	40	19.533	1.361	52
60°	11.131	1.590	30	11.431	1.699	31	12.114	1.443	33	13.515	0.895	36
90°	8.363	0.982	22	7.405	1.126	20	9.508	1.040	26	12.589	0.812	34

*AVG CA* average contact area, *SD* standard deviation, *PCA* percentage contact area

**Table 2:** Average contact area and percentage contact area when using the round-tip catheter (mm^2^).

Contact angle	Contact force (gf)											
	2			4			6			10		
	AVG CA	SD	PCA (%)	AVG CA	SD	PCA (%)	AVG CA	SD	PCA (%)	AVG CA	SD	PCA (%)
0°	6.365	0.508	25	8.730	0.840	34	9.039	1.040	35	10.325	1.159	40
30°	6.055	1.075	24	6.999	0.825	27	9.982	0.768	39	12.579	1.107	49
45°	3.592	0.493	14	4.906	0.421	19	5.547	0.629	22	6.815	0.898	27
60°	3.699	0.724	14	5.869	0.719	23	6.782	0.821	26	8.222	0.625	32
90°	3.829	0.294	15	6.309	0.320	25	7.797	1.134	30	7.990	0.553	31

Contact angle	Contact force (gf)											
	15			20			30			40		
	AVG CA	SD	PCA (%)	AVG CA	SD	PCA (%)	AVG CA	SD	PCA (%)	AVG CA	SD	PCA (%)
0°	11.779	1.292	46	12.343	1.042	48	13.661	0.852	53	15.578	1.274	61
30°	14.417	2.141	56	13.697	1.857	53	17.693	1.394	65	16.788	0.581	65
45°	8.123	0.652	32	9.704	0.841	38	12.730	1.583	50	13.725	1.954	53
60°	9.354	0.811	36	10.275	0.868	40	10.464	1.740	41	14.310	2.286	56
90°	8.535	0.751	33	9.340	0.638	36	10.357	1.039	40	11.914	0.909	46

*AVG CA* average contact area, *SD* standard deviation, *PCA* percentage contact area

$$\mathrm{Percentage}\, \mathrm{contact}\, \mathrm{area} = \left(\frac{\mathrm{Catheter}/\mathrm{heart}\, \mathrm{contact}\, \mathrm{area}}{\mathrm{Catheter}\, \mathrm{tip}\, \mathrm{surface}\, \mathrm{area}}\right)\times 100,$$

where the catheter tip surface area of the flat-tip catheter is 37.26 mm^2^ and that of the round-tip catheter is 25.67 mm^2^.

The standard SI unit for force is the Newton (N), but gram-force (gf) is frequently used to measure contact force in the field of catheter ablation research (1 gf = 0.00981 N, which is the force acting on a mass of 1 g under the Earth’s gravitational acceleration of 9.81 m/s^2^). The contact forces tested in this study were 2, 4, 6, 10, 15, 20, 30, and 40 gf, which correspond to 0.0196, 0.0392, 0.0588, 0.098, 0.147, 0.196, 0.294, and 0.392 N, respectively.

**Figure 5:**
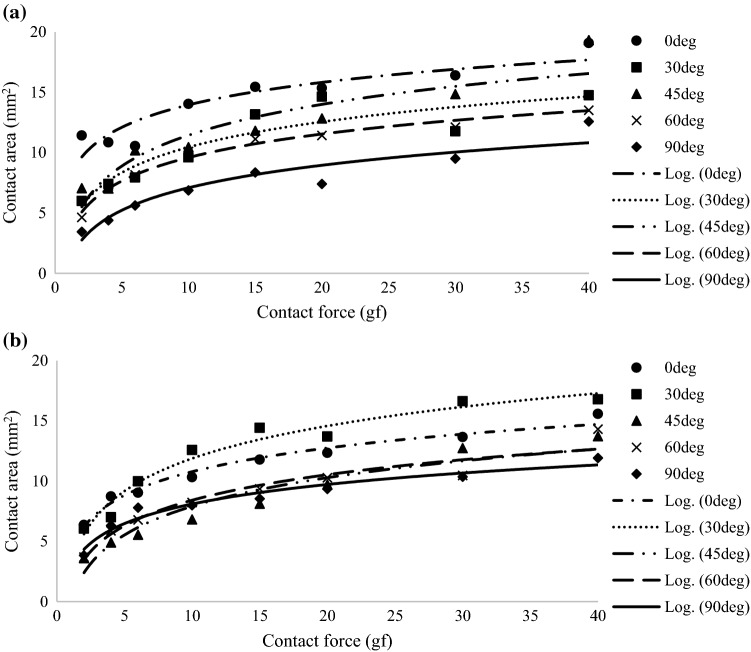
Plot of average contact area and contact force for (**a**) flat-tip catheters and (**b**) round-tip catheters at each contact angle.

Figure 5 shows a plot of the catheter contact areas created with the contact forces on the *x*-axis and the contact area on the *y*-axis for both catheter shapes. This plot illustrates the correlation between contact force, contact angle, and contact area. The results revealed a positive correlation between contact force and contact area, in which increased contact force was associated with increased contact area. Moreover, the contact angle had as strong an effect on the contact area as the contact force did. At the same contact force, the correlation between contact angle and contact area was inverse; that is, a smaller contact angle was associated with an increased contact area. The logarithmic approximation formulas for expressing the relationship between contact force and contact area for each catheter contact angle are shown in Table 3. The data reveal that the correlation between contact force and the contact area is a logarithmic function with R-squared (*R*^2^) being nearly equal to 1.

**Table 3:** Approximation formulas expressing the relationship between catheter contact force and contact area for each catheter contact angle, where *x* is catheter contact force (gf), *y* is catheter contact area (mm^2^), and *R*^2^ is coefficient of determination, respectively.

Angle (°)	Flat-tip		Round-tip	
	Approximation formula	*R*^2^	Approximation formula	*R*^2^
0	*y* = 2.685ln(*x*) + 7.782	0.837	*y* = 2.837ln(*x*) + 4.251	0.974
30	*y* = 3.036ln(*x*) + 3.465	0.845	*y* = 3.903ln(*x*) + 2.890	0.961
45	*y* = 3.689ln(*x*) + 2.952	0.867	*y* = 3.429ln(*x*) + 0.012	0.926
60	*y* = 2.807ln(*x*) + 3.137	0.984	*y* = 3.062ln(*x*) + 1.363	0.936
90	*y* = 2.693ln(*x*) + 0.892	0.893	*y* = 2.341ln(*x*) + 2.709	0.953

**Figure 6:**
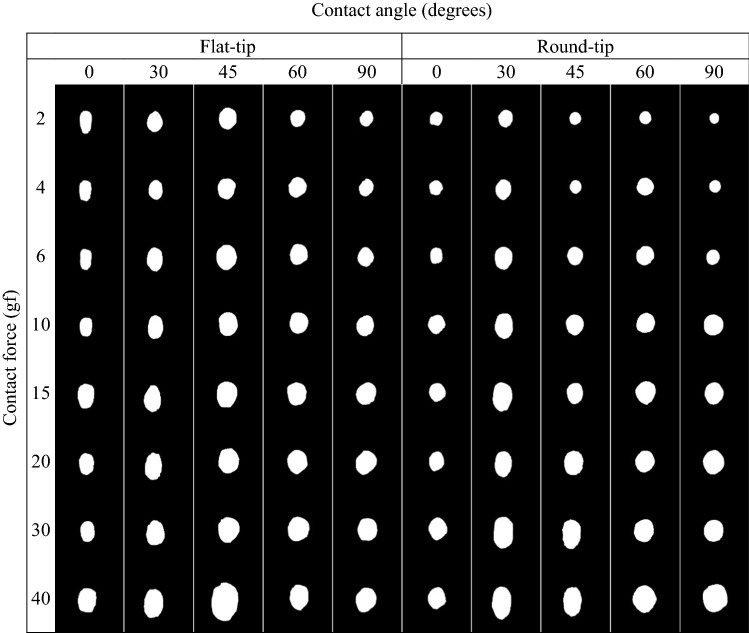
Average contact area morphology using flat-tip and round-tip catheters under various conditions.

Figure 6 shows binarized images of the average contact areas under the various conditions (8 contact forces and 5 contact angles) when using the flat-tip and round-tip catheters. These data clearly show that the contact angle had as much influence as the contact force on the contact area. For example, using the flat-tip catheter at a contact angle of 90°, the contact force increased from 2 to 4, 6, 10, 15, 20, 30, and 40 gf, and the average contact area increased from 3.445 to 4.392, 5.627, 6.879, 8.363, 7.405, 9.508, and 12.589 mm^2^, respectively. In addition, using the round-tip catheter at a contact angle of 90°, the contact force increased from 2 to 4, 6, 10, 15, 20, 30, and 40 gf, and the average contact area increased from 3.829 to 6.309, 7.797, 7.990, 8.535, 9.340, 10.357, and 11.914 mm^2^, respectively. Similar trends were seen for both shapes of catheters at contact angles of 60°, 45°, 30°, and 0°.

## Discussion

### Major Findings

Our major findings are as follows: (i) the morphology of the contact area can be divided into four types: rectangular, circular, ellipsoidal, and semi-ellipsoidal. The morphology of the contact area indicates that (ii) the correlation between contact force and contact area is a logarithmic function; that is, increased contact force was associated with increased contact area, and the contact angle has as strong an effect on the contact area as contact force does. (iii) There is an inverse correlation between contact angle and contact area; smaller contact angle is associated with increased contact area.

### Morphological Characterization of the Contact Area

**Figure 7:**
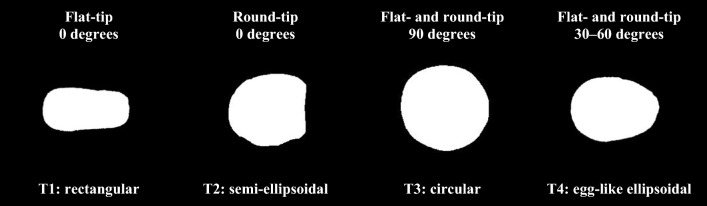
Contact area morphologies. The T1 morphology represents the contact area of the flat-tip catheter at a contact angle of 0°. The T2 morphology represents the contact area of the round-tip catheter at a contact angle of 0°. The T3 morphology represents the contact area of both the round- and flat-tip catheters at a contact angle of 90°. The T4 morphology represents the contact area of both the round- and flat-tip catheters at a contact angle of between 30° and 60°.

To elucidate the effects of catheter contact angle and contact force on the contact area, we constructed a heart muscle surface flattener to maintain a flat surface to test a range of contact angles. This instrument was designed to achieve improved experimental reproducibility. Although in routine clinical ablation procedures, the surface of the heart tissue is not flat, our data clearly demonstrated that the contact angle and shape of the catheter tip substantially affected the contact area morphology. In summary, we categorized the morphology of the contact area into four types, as shown in Fig. 7. A notable difference occurred when the catheter angle became parallel to the heart surface. Contact area morphology became rectangular when using a flat end tip and semi-ellipsoidal when using a round end tip. This observation clearly shows the effect of the shape of the catheter. When contact is made at a perpendicular angle, the contact area morphology is circular because the projected area of both catheters is a circle, and thus the contact area becomes circular. When the catheter is inclined, the contact area becomes ellipsoidal like an egg. Those morphological character trends changed similarly for both round- and flat-tip catheters except in the parallel (0°) direction.

### Correlation Between Contact Force and Contact Area

Catheter contact force shows a strong positive correlation with contact area. When the contact force was increased, the contact area also increased. These results are similar to those in earlier reports^1^,^3^–^5^,^22^,^27^; however, it is essential to consider the small changes in contact area that occurred at higher contact forces. The contact area increased monotonically but logarithmically. The slope of the graph changes slightly when the contact force is between 15 and 40 gf, which is in contrast to the greater change in slope when during initial contact when the contact force ranges from 2 to 15 gf. The equation for estimating contact area might help those performing this procedure to understand the relationships among the parameters and to calculate the contact area as a function of contact force at each contact angle. Our data suggest a limit to the extent by which lesion size can be increased by increasing the contact force. The catheter contact angle relative to the heart muscle tissue surface can also needs to be considered when calculating the desired lesion size.

### Correlation Between Contact Angle and Contact Area

The results clearly demonstrate that the contact angle is a key determinant of the contact area morphology. In addition, the contact angle substantially affects the contact area of the catheter tip regardless of the contact force. For the flat-tip catheter, the minimum contact area was produced at a contact angle of 90° and increased with decreasing contact angle from 90° to 60°, 45°, 30°, and 0°. For the round-tip catheter, the minimum contact area was produced at a contact angle of 90° and increased with decreasing contact angle from 90° to 60°, 45°, 0°, and 30°. For both catheter shapes, the contact area progressively increased when the contact angle was decreased. However, our results show a difference between the flat- and round-tip catheters at 0° and 30°. These differences were due to the difference in shape and size between the two shapes of catheter tip. The two catheters used for this study were made by different manufacturers and differ in size according to their shape, especially at a contact angle between 0° and 30°. The round-tip catheter makes less surface contact with the heart tissue surface compared with the flat-tip catheter. Despite this fact, the results of the experiment as a whole show a similar tendency.

### Clinical Implications

Our data should be useful for those performing this procedure to understand the relation among the parameters and plan their treatment strategy beforehand. From our experiments, the contact area morphology was derived as a function between the contact angle and contact force. It is reasonable to assume that the contact area is directly related to the area of resulting lesion.

### Study Limitations

This study has several limitations. First, we aimed to test our assumptions about contact angle, contact force, and contact area by using two different shapes of commercially available catheter tips. The two catheters were made by different manufacturers, and thus have some differences in design. Accordingly, we did not compare the differences in results between the catheters. Second, to provide better reproducibility of our *in vitro* experiments, we developed a special instrument that precisely adjusts the catheter angle between the catheter tip and the heart muscle. The instrument consists of a heart muscle surface flattener and a catheter-tip-angle setter. In clinical practice the shape of the heart tissue surface varies according to the part of the heart, and thus the catheter tip orientation can rarely be optimized due to restricting structures such as trabeculated muscle, valves, or the papillary muscle. Nevertheless, at the present stage of research on catheter ablation (pre-clinical experiment studies), it necessary to perform tests on flat surfaces to clearly demonstrate the specific effects of the catheter contact angle and contact force on the contact area of the heart tissue surface. Lastly, to produce effective ablation lesions, the depth of the lesion is at least as important as the ablation size. In this study, we did not investigate whether the catheter contact angle and contact force affected the depth of the ablation lesion; however, we conducted experiments to elucidate the effect of the catheter contact angle and contact force on the contact area. Our findings might be validated in the near future through numerical simulations such as the Finite Elemental Method, which can be used to estimate cardio-muscular deformation in response to catheter tip contact or a practical investigation through an *in vitro* heart muscle ablation experiment.

## Conclusion

This study clearly demonstrated a substantial impact of the contact angle and contact force of a catheter on the size and morphology of the contact area in catheter ablation procedures. The contact area should be directly related to the lesion area. Our data may help doctors understand the relationships among contact angle, contact force, and contact area in ablation therapy procedures. Such information should help doctors plan appropriate treatment strategies in consideration of each patient’s conditions.
